# Hepatitis B Virus and microRNAs: A Bioinformatics Approach

**DOI:** 10.3390/ijms242417224

**Published:** 2023-12-07

**Authors:** Verdiana Zulian, Giulia Fiscon, Paola Paci, Anna Rosa Garbuglia

**Affiliations:** 1 Virology Laboratory, National Institute for Infectious Diseases “Lazzaro Spallanzani” IRCCS, 00149 Rome, Italy; verdiana.zulian@inmi.it; 2 Department of Computer, Control and Management Engineering, Sapienza University of Rome, 00185 Rome, Italy; fiscon@diag.uniroma1.it (G.F.); paci@diag.uniroma1.it (P.P.); 3 Institute for Systems Analysis and Computer Science “Antonio Ruberti”, National Research Council, 00185 Rome, Italy

**Keywords:** microRNA, bioinformatics tools, biomarkers, hepatitis B virus, infectious diseases, chronic infection, antiviral treatment

## Abstract

In recent decades, microRNAs (miRNAs) have emerged as key regulators of gene expression, and the identification of viral miRNAs (v-miRNAs) within some viruses, including hepatitis B virus (HBV), has attracted significant attention. HBV infections often progress to chronic states (CHB) and may induce fibrosis/cirrhosis and hepatocellular carcinoma (HCC). The presence of HBV can dysregulate host miRNA expression, influencing several biological pathways, such as apoptosis, innate and immune response, viral replication, and pathogenesis. Consequently, miRNAs are considered a promising biomarker for diagnostic, prognostic, and treatment response. The dynamics of miRNAs during HBV infection are multifaceted, influenced by host variability and miRNA interactions. Given the ability of miRNAs to target multiple messenger RNA (mRNA), understanding the viral–host (human) interplay is complex but essential to develop novel clinical applications. Therefore, bioinformatics can help to analyze, identify, and interpret a vast amount of miRNA data. This review explores the bioinformatics tools available for viral and host miRNA research. Moreover, we introduce a brief overview focusing on the role of miRNAs during HBV infection. In this way, this review aims to help the selection of the most appropriate bioinformatics tools based on requirements and research goals.

## 1. Introduction

Hepatitis B virus (HBV) is a small double-stranded DNA virus of approximately 3.2 Kilobases (kb) in pairs. In the nucleus, the relaxed circular DNA (rcDNA) is converted in covalently closed circular DNA (cccDNA), that is the template for HBV gene products, including pregenomic RNA (pgRNA) [1], and mRNAs encoding several proteins: hepatitis B surface antigen (HBsAg), hepatitis B core antigen (HbcAg), x protein (HBx), and HBV polymerase [2]. Moreover, the S gene is preceded by two regions, called preS1 and pre-S2, that encode for preS2-S protein (middle protein) and preS1-preS2-S protein (large protein). cccDNA could be considered a stable minichromosome in non-dividing hepatocytes, and it is the main factor of HBV persistence also in patients under viral therapy [3,4]. HBV belongs to the *Hepadnaviridae* family, genus *Orthohepadnavirus* [5].

Ten HBV genotypes (A-J) and forty subtypes have been identified [6]. The genetic difference among genotypes is 10% and 4% among subtypes. HBV infection could have a different clinical outcome. In recovered infections, the HbsAg (the first structural protein detected in the serum of patients with acute primary infection) disappears, and anti-HbsAb seroconversion represents the main indication of recovery [7]. If HbsAg persists for more than six months, a chronic infection is established. However, HBV DNA can persist in 0.1–0.6% of HBV infections in the absence of HbsAg [8]. Chronic hepatitis B infection (CHB) is developed in 5% of HBV-infected patients. The World Health Organization (WHO) estimates that in 2019, 296 million people world-wide were living with CHB (https://www.who.int/news-room/fact-sheets/detail/hepatitis-b, accessed on 15 July 2023). Without any therapy, 15–20% of patients with CHB could progress towards cirrhosis. The risk to progress towards chronic infection is inversely proportional to age at infection; approximately 90% of CHB infections occur in newborns, 20% in children, and less than 5% in immunocompetent adults [9,10,11]. A recent study attributed 331,000 deaths to HBV-related cirrhosis and chronic diseases [12,13].

The main antiviral therapy used in CHB treatment are Nucleos(t)ide analogues and pegylated interferon (Peg-IFNα). Six nucleos(t)ide analogues (NUCs) have been approved for oral use; however, Entecavir, TDF (disoproxilfumarate tenofovir), and tenofovir alafenamide are the main drugs used since they show a low risk of viral resistance and higher antiviral activity in comparison to the former nucleos(t)ide analogues: lamivudine, adefovir, and telbivudine [9]. These therapies have as target the viral polymerases, and their administration leads to an HBV DNA suppression, but the cccDNA are poorly affected by this therapy, thus the time of treatment is very long, and only a small average of treated patients reach the anti-HbsAg conversion [14]. For example, only 3–5% of patients treated with TDF lose HbsAg with an anti-HBs seroconversion after 10 years of antiviral therapy even though the HBV DNA level falls under 10 IU/mL after 48 weeks of treatment [14,15,16].

Peg-IFNα interferes with the entire process of HBV replication and is able to promote the degradation of cccDNA, but only 5% of patients under Peg-IFNα therapy reach anti-HBs seroconversion at week 48 of treatment. Moreover, the side effects observed during the Peg-IFNα administration, such as alopecia, insomnia, anxiety, and thyroid dysfunction, limit the use of this antiviral compound [17]. New antiviral drugs that have different targets of viral cycle or specific viral proteins are being used in clinical trials. For example, Myrcludex (bulevirtide), that interferes with the viral entry in the hepatocyte, or Zm_H150, that interferes with the HBV capsid activity [18].

Given the low percentage of patients who achieve seroconversion from HbsAg to anti-HBs even after years of NUCs therapy (5–7%) [19], there is debate about the need to stop antiviral treatment, at least in patients who seem to benefit from the administration of antivirals and where a viral rebound seems to be remote. Nevertheless, the criteria for therapy discontinuation are still to be established/defined [20]. Clinical trials on patients who were non-cirrhotic patients but were still HbsAg-positive and discontinued antiviral therapy showed that 1.2% had severe reactivation of hepatitis, 0.37% dead, or they needed liver transplantation [17].

Several biomarkers have been proposed to better understand the efficacy of anti-HBV therapy: cccDNA, HBV RNA, and HbcrAg.

1. cccDNA. The eradication of cccDNA indicates complete cure [21], while the term sterilization is used to defines the clearance of integrated DNA; they are not generally reached with the actual therapies. cccDNA should be quantified in liver tissue. Since nonstandard methods are available for this analysis, cccDNA quantification is still considered a research tool [22].
2. HBV RNA represents a biomarker that indirectly measures the activity/presence of cccDNA. Nevertheless, the available assays cannot distinguish between HBV RNA produced by cccDNA and integrated HBV DNA or that from spliced RNA. The HBV RNA measurement has low sensitivity, and interference with HBV DNA has been described [23]. There are still few data on HBV RNA prediction of liver-related complications, including cirrhosis or HCC [24]. A retrospective study of 96 HbeAg-negative patients with undetectable HBV DNA under Nas reported that HBV RNA was detectable in 52% of patients at years 4 and 5% at 10–14 years of follow-up [25].
3. HB core-related antigen (HbcrAg) includes three proteins: hepatitis B core antigen (HbcAg), HbeAg, and p22-p22cr derived exclusively from cccDNA transcriptional activity [1]. A high HbcrAg level seems to be associated with a higher hazards ratio of incident HCC (5–6.3-fold) [26,27]. However, a cut-off to distinguish the patients with a worse prognostic value is lacking. Its level seems to predict viral relapse after NUCs therapy interruption; however, the sensitivity of the assay for HbcrAg detection should be improved [19].

From this overview, it is clear that the possibility that there is an eradication of the infection or sterilization through therapeutic treatment is low (1–4%) in patients treated with NAS. It is relevant to identify some biomarkers able to indicate whether the antiviral treatment has provided a benefit to liver status or whether it is predictive of the absence of negative effects linked to antiviral treatment interruption. The mentioned biomarkers (cccDNA, HBV RNA, and HbcrAg) have strong limitations due to lack of specificity, and sensitivity and do not give information on liver status, but only on the presence of HBV DNA during treatment [28].

In recent decades, there has been an increase in the study of microRNAs (miRNAs), given their importance in the regulation of gene expression, working as switches [29]. In fact, it has been found that miRNAs can regulate a wide range of biological processes, including development, cell proliferation, differentiation, apoptosis, metabolism, and metastasis [30,31]. Thus, the aberrant expression of miRNAs has a crucial impact on various pathological conditions, such as cardiovascular diseases, neurological disorders, immune response, cancer, and viral infections [32,33,34,35,36]. It has been shown that miR-1 and miR-133 were deregulated in human heart failure; miR-24, miR-95, miR-199a, and miR-214 were upregulated both in cardiac hypertrophy and end-stage failing human hearts, for example. Meanwhile, the miR-20a family may promote the development of Alzheimer’s disease by targeting the Amyloid Precursor Protein (APP) [32]. In addition, some miRNAs have been detected in biological fluids, i.e., serum, plasma, and urine. These miRNAs, called circulating miRNAs, are stable and easily detectable using non-invasive methods. It is for these reasons that circulating miRNAs have great potential as biomarkers [37]. For instance, miR-152-3p and miR-155 have been associated with prostate cancer, miR-19b and miR-21 with lung cancer, miR-1 with cardiovascular disease, and miR-151-3p with human immunodeficiency virus (HIV) [38,39].

In light of the above, it is plain to see that miRNAs may have several clinical implications, ranging from diagnosis and prognosis to treatment development and personalized medicine [40,41,42]. Therefore, in the context of HBV infection, miRNAs may also be considered promising biomarkers because they can be associated with liver health status, the inflammatory process, and the level of HBV replication [43,44,45,46].

In view of the complexity of this field of study, the combination of bioinformatics and experimental validation plays an important role in miRNAs research and analysis.

In this work, we describe several bioinformatics tools and algorithms available for miRNA research, including miRNA identification, target prediction, enrichment analysis, and miRNA–disease associations, especially in virus infections. Moreover, we introduce a brief overview about the role of miRNAs during HBV infection. In this way, this review could aid the researcher who wants to explore the miRNAs and bioinformatics world associated with viral diseases.

## 2. microRNAs Biogenesis

The first miRNA to be identified was lin-4 in *C. elegans* in 1993 [47,48]. Subsequent studies revealed that lin-4 was a small non-coding RNA of approximately 22 nucleotides (nt) in length, which acted as a post-transcriptional regulator [20]. Since then, numerous miRNAs have been identified in various organisms, such as humans, plants, animals, and some viruses [49,50,51,52,53,54]. 

The canonical biogenesis pathway of miRNAs begins in the nucleus, with the transcription from DNA into a primary miRNA (pri-miRNA) by RNA polymerase II (RNA pol II) or III [55]. The pri-miRNA consists of a hairpin structure, which may contain from one to six miRNA precursors. The pri-miRNA is then processed by a complex of Drosha/DiGeorge syndrome (DGCR8), which cleaves it into a precursor miRNA (pre-miRNA). The pre-miRNA typically consists of approximately 70 nt, with a characteristic stem–loop structure. The pre-miRNA is subsequently exported from the nucleus to the cytoplasm by Exportin-5 [56]. In the cytoplasm, the pre-miRNA is cleaved by Dicer by removing the terminal loop; the result is a miRNA duplex. One strand of the mature miRNA, known as the guide strand, is loaded into the RNA-induced silencing complex (RISC) containing Argonaute (AGO) protein. The guide strand guides the RISC complex to its target mRNA, leading to the regulation of gene expression. Other non-canonical pathways of miRNA biogenesis have recently been discovered, such as “Drosha-independent” and “Dicer-independent” [57].

Therefore, miRNAs are among the main actors of the post-transcriptional gene silencing through binding to messenger RNA (mRNA) molecules of target genes, usually in the 3′ untranslated region (3′-UTR) by base pairing with nucleotides 2–7 of the miRNA 5′-end, called *seed-sequence*. The degree of complementary between miRNA and its target mRNA influences the regulatory action of miRNAs. A perfect complementarity leads to mRNA degradation, while an imperfect complementarity inhibits its translation into a protein, thus reducing the expression of the target gene [58]. However, recent studies have shown that miRNAs can also interact with different regions of their target mRNAs, including coding sequence (CDS), and 5′-UTR [29]. It is also important to mention that a given miRNA can bind to several target mRNAs, and a single mRNA can be targeted by different miRNAs [59].

Another interesting aspect involving miRNAs is the mechanism of post-transcriptional regulation, known as the ceRNA mechanism or “miRNA sponges” [60]. These miRNA sponges are RNA molecules containing multiple binding sites for a specific miRNA. Consequently, the miRNA activity is inhibited, and this interference can increase the expression of related target genes [61]. miRNA sponges can be protein-coding and non-coding RNAs [62], including the new-acknowledged class of circular RNAs (circRNAs). CircRNAs are nucleic acid molecules that have a closed-loop RNA form [63,64] and have been identified in humans and other species, but currently not in viruses [65,66]. The identification of the circRNA-miRNA-mRNA regulatory axis is an ongoing research area.

In recent years, several viruses have been found to encode viral microRNAs (v-miRNAs), such as EBV, KSHV, HPV, HSV, MCPyV, HIV, and HBV [67,68,69,70]. At present, a large number of v-miRNAs have been predicted computationally (e.g., SARS CoV-2, HAV, and Nipah [71,72,73]), but only a small part of them have been validated experimentally [74,75]. V-miRNAs are transcribed from viral genomes and their biogenesis is similar to their host, for instance, HBV-miR-3 [69]. These v-miRNAs are expressed during viral infection and are able to manipulate host and viral gene expression to their advantage [76,77].

## 3. Bioinformatics Tools for microRNAs Analysis

The advancement of high-throughput sequencing technologies, also known as next-generation sequencing (NGS), led to the rapid generation of massive biological data [78,79]. Therefore, bioinformatics comes from the need to manage, analyze, and interpret this vast amount of data.

Bioinformatics tools developed for miRNA study can help to understand the regulatory roles of miRNAs in various biological processes and diseases via curated miRNA databases, the identification of known and novel miRNAs, target prediction, functional annotation, miRNA-mRNA interaction networks, expression analysis, and disease association studies.

Due to the lack of a single comprehensive tool available for miRNA analysis, the use and combination of different tools is necessary.

In recent years, new tools are constantly developed, especially after the integration of artificial intelligence (AI) techniques. Machine learning (ML) algorithms are mainly used to identify novel miRNAs [80], predict their gene targets [81], and model regulatory networks [82].

In order to facilitate the choice of the most appropriate bioinformatic tools according to the individual needs and research objectives, we present an overview of the main bioinformatics tools devoted to the computational analysis of miRNAs, with a particular focus on viral and host miRNAs (summarized in Table 1, Table 2, Table 3 and Table 4 and deeply discussed in the next sections).

As discussed, the bioinformatic resources currently available for viral and host miRNAs analysis and their interactions are incomplete. To achieve a thorough analysis, the incorporation of non-specific tools is essential. This raises new challenges in bioinformatics applied to virology.

Most of the tools described are web-based and designed to be user-friendly, where one of the best ways to access information of interest is by utilizing the search and browse buttons in the homepage of the website. These computational tools could provide a first step for further explorations, and experimental validations are able to manipulate host and viral gene expression to their advantage [76,77].

### 3.1. microRNA Databases

In recent years, thousands of miRNAs have been discovered and identified in several species [74]. Consequently, it is crucial to store data related to miRNA sequences, target genes, expression profiles, annotations, and functional information. miRNA databases allow these data to be organized and categorized, making it easier to access and use for users, e.g., NCBI [83], GEO [84], RNAcentral [85], plasmiR [86], and miRBase [87,88,89,90] (Table 1).

To date, there are numerous human miRNA databases that provide curated and controlled information, and some of these include multiple basic tools [91]. Moreover, analysis bioinformatic tools can also serve as databases, particularly those specialized in target prediction [92,93,94] and miRNA–disease associations [95,96]. However, there are relatively fewer repositories that have been specifically developed for v-miRNAs and virus–host interactions during viral infections, such as VIRmiRNA [97], AntiVIRmiR [75], and VIRBase v3.0 [98].

The NCBI Viral genomes resource [83] is a reference specialized database of the National Center for Biotechnology Information (NCBI). In this database, it is possible to find specific RefSeq records and validated genome sequences (e.g., the number of Hepatitis B virus genomes is 6058) knowing that the viral genome sequence is essential for the identification of novel v-miRNAs by predicting their precursors (pre-miRNA sequences) [99] and identifying putative mature miRNAs [71].

In GenBank, there is only one miRNA encoded by HBV, i.e., HBV-miR-3 [69]. The GenBank accession number for the HBV-miR-3 is KY684291.

The GEO (Gene Expression Omnibus) [84] database is another database provided by NCBI which serves as a repository for high-throughput gene and miRNA expression data. It allows for miRNA expression data in virus-infected cells to be downloaded. These types of data can be used to study the different expression patterns of host miRNAs in the context of specific viral infections.

RNAcentral [85] is a user-friendly and web-based centralized resource of various types of RNA, including mRNAs, miRNAs, long non-coding RNAs (lncRNAs), ribosomal RNAs (rRNAs), transfer RNAs (tRNAs), and small nuclear RNAs (snRNAs), by including fifty-one specific databases. This database provides information about sequences, secondary structures, annotations, expression profiles, miRNA–target, and miRNA-lncRNA interactions. The search can be performed by gene, species, accession number, name, or sequences [100]. 

The use of miRNAs as a biomarker is one of the main goals of the research on miRNAs. Therefore, the plasmiR [86] database has been developed to explore the role of circulating miRNAs as experimentally validated diagnostic and prognostic biomarkers. The database provides the possibility to search by miRNAs and/or diseases. In addition, expression, sample, and biomarker types can be added as search filters.

miRBase [87,88,89,90] is the most used database that serves as a central repository for miRNA sequences and annotations. It offers a curated collection of experimentally validated sequences and related information from different species (271 organisms in the last version V22) [74]. However, the quantity of v-miRNA sequences is restricted. As of the time of writing this work, no HBV-miRNA is present in this database.

Within the database, it is also possible to find information about hairpin precursor miRNAs (pre-miRNA), literature references, identification methods, and linking out to other resources. Furthermore, miRBase provides guidelines on miRNA annotation and nomenclature [101,102]. In addition, it is commonly used as the first step in microRNA sequencing (miRNA-seq) data analysis through sequence alignment [89,103,104].

Few specific resources are then available for viral miRNAs and their targets. VIRmiRNA [97] has been the first repository dedicated to experimentally validated v-miRNAs, their targets, and antiviral host miRNAs. Therefore, the VIRmiRNA database has three different categories: VIRmiRNA, VIRmiRtar, and AVIRmiR.

The VIRmiRNA subdatabase shows information about v-miRNA, including miRNA name, sequence, length, GC content, experimental methods used, target, and reference. VIRmiRtar contains v-miRNA targets; therefore, the gene, target region, reference, cell line, and experimental methods used are shown. Furthermore, the associated network interaction is displayed. The AVIRmiR section allows for the interplay between virus and host during infection to be investigated because the host miRNAs could influence and modulate various aspects of the infection [105]. The easy-downloadable information about these host miRNAs includes name, sequence, target, target region, experimental methods, and reference. Additionally, the Seed-Align (miRBase and VIRmiRNA), BLAST, TarFind, and Map tools are directly available on the website. VIRmiRNA is an accessible web tool and can be accessed, but it has not received any updates since 2014, due to its replacement with AntiVIRmiR [75]. In this new version, the subdatabases VIRmiRNA, VIRmiRtar, and AVIRmiR have been updated and replaced by VIRmiRNA2, VIRmiRTar2, and AntiVmiR, respectively. Moreover, a fourth subcategory has been added, DEmiRVIR. DEmiRVIR includes the differentially expressed miRNAs of hosts and viruses. Therefore, it is possible to choose the virus of interest directly from the DEmiRVIR page. The abovementioned bioinformatics tools BLAST, TarFind, and Map are also available in this version, as well as Cytoscape v3.10.1 [106] that can be used to visualize and analyze interaction networks.

To explore the virus–host interaction in terms of ncRNA, VIRBase v3.0 [98] is a comprehensive online repository, which collects experimental and predicted data from 116 viruses and 36 host organisms, human included. To date, 283 from HBV and 156 from HCV interactions are stored. The search can be performed by miRNA name, virus name, and RNA category. It is also possible to select the host organism and the interaction type: Host–Host (HH), Host–Virus (HV), Virus–Host (VH), and Virus–Virus (VV), as well as the detection method: computational prediction, strong, and weak experimental evidence.

The user can query the database by interaction type, detection method, or organism and easily download all the results. In particular, the downloaded file contains the virus and host name, taxonomy ID, reference, a confidence score, and information about the two interactors (virus and/or host) [98]. Moreover, two prediction web tools are available, InstaRNA and PRIdictor. The first tool can be used to predict interactions between two RNA sequences, while the second is for protein–RNA interactions.

In addition, there is lots of virus bioinformatics research freely available, but not specifically about miRNAs (i.e., ViralZone [107], HBVdb [108], BV-BRC [109], and ViMC [110]). HBVdb [108] is a specialized database in hepatitis B virus (HBV) that contains curated and annotated data on viral genome sequences, genetic variations, protein structures, and others. The website provides online tools for sequence analysis and genotyping. Similar to HBVdb, BV-BRC [109] contains several resources on viruses and bacteria.

ViMIC [110] allows information to be obtained on curated virus mutations, integration sites in the host genome, and cis-effects. The target gene features and data of virus-related diseases are also included on the website. The target gene feature provides the curated storage of target genes influenced by viral genome insertion or virus gene/protein/region regulation. All information is easily accessible and clearly arranged by browsing on the website.

**Table 1:** Overview of described microRNA databases.

Database/Tool Name	Summary Features	Last Update	Link	Ref.
NCBI Viral Genomes Resource *	RefSeq virus dataset. Total validated viral genome sequences. miRNAs expression data in virus-infected cells. Experimentally validated.	2023	https://www.ncbi.nlm.nih.gov/genome/viruses/ (accessed on 10 April 2023)	[83]
GEO	Gene expression data. miRNA expression data. Experimentally validated.	2023	https://www.ncbi.nlm.nih.gov/geo/ (accessed on 12 May 2023)	[84]
RNAcentral	miRNA sequences. Pre-miRNA sequences. Annotations. miRNA expression profiles. miRNAs target gene.	2023	https://rnacentral.org (accessed on 10 May 2023)	[100]
plasmiR	Circulating miRNAs as diagnostic and prognostic biomarkers. Experimentally validated.	2021	https://dianalab.e-ce.uth.gr/plasmir/#/ (accessed on 18 June 2023)	[86]
miRBase	miRNAs sequence. Pre-miRNAs sequence. Annotations. Sequence alignments.	2022	https://www.mirbase.org (accessed on 5 April 2023)	[74]
VIRmiRNA *	VIRmiRNA: Viral miRNAs sequence. VIRmiRtar: Viral miRNAs target gene. AVIRmiR: Antiviral host (human) miRNAs.	2014	http://crdd.osdd.net/servers/virmirna/ (accessed on 7 July 2023)	[97]
AntiVIRmiR *	DEmiVIR: Host/Viral miRNAs regulation (up/down). AntiVmiR: Antiviral host (human) miRNAs. VIRmiRNA2: Viral miRNAs sequence. VIRmiRTar2: Viral miRNAs target gene. Experimentally validated.	2022	https://bioinfo.imtech.res.in/manojk/antivirmir/ (accessed on 23 June 2023)	[75]
VIRBase v3.0 *	Virus–host ncRNA interaction. Experimentally validated and computationally predicted.	2021	http://www.virbase.org (accessed on 28 May 2023)	[98]

* Viral specific database/tool.

### 3.2. microRNA Identification and Annotation

Identification refers to known and novel miRNAs. As a preliminary step for the identification and annotation of known/novel miRNAs, miRNA sequences can be directly searched inside a database [75,83,111,112] where known miRNAs are annotated.

The miRNAs annotation process allows specific information and properties to identify miRNA, including genomic location, precursor structure (pre-miRNA), mature sequence, evolutionary conservation across species, target gene, and biological function, to be assigned [101]. The identification of novel miRNAs involves several steps that include both computational and experimental approaches. Computational algorithms and tools are employed to predict putative miRNA candidates from sequencing data, followed by experimental validation methods to confirm them.

As a result of the discovery of the first v-miRNA in EBV [53], only a small part of v-miRNAs has been identified. As discussed previously, only two miRNAs encoded by HBV have been discovered, i.e., HBV-miR-3 and HBV-miR-6 [70,113].

The experimental identification of novel miRNAs is complex and expensive, especially of v-miRNAs in host-infected cells. Therefore, bioinformatic tools could aid to narrow down the search and select potential mature miRNAs in silico [72,73]. Currently, bioinformatics tools and algorithms have been developed considering the specific characteristics of miRNAs [114], such as sequence conservation, hairpin structures from a pre-miRNA sequence [115], and thermodynamic stability [116] (Table 2). 

In general, the first and most important step to identify novel miRNAs is by predicting the secondary structure of the pre-miRNAs from the whole genome of interest, and then the putative mature miRNAs can be extracted from the pre-miRNA sequences [117].

Computational tools for pre-miRNAs prediction may be classified into two main categories: homology/comparative [118] and ab initio methods [119].

A homology-based approach compares putative pre-miRNAs to known pre-miRNAs from databases and/or the literature. This method uses information from homology sequences and structures [118], involving sequence alignment to search similarities. miRAlign [120], miRExpress [121], and miRseeker [122] are some homology-based tools.

However, the comparative genomic approach uses the evolutionary conservation of some pre-miRNAs via multiple alignments between related species. Therefore, conserved pre-miRNA structures are searched based on specific characteristics of their stem–loop structures [123]. MiRscan [124] is a web server tool and one of the computational tools that uses the comparative genomic method. The algorithm implements a probabilistic method and it is trained by drawing information from conserved instances derived from two closely related species [125]. The criteria used are a set of characteristic features, such as 5′ and 3′ conservation, complementary base pairing, distance from the loop, and bulge symmetry. Homology and comparative approaches may require additional filters, e.g., minimum free energy (MFE) and position of mature miRNA within the hairpin structure, to increase specificity [115].

In the case of viral miRNAs, both the described methods are inappropriate due to the considerable diversity of viral genomes and the small amount of data available [126,127]. Therefore, the ab initio approach is the most commonly used method to predict viral pre-miRNAs, such as RNAFold [128], mFold [99], Vir-Mir db [129,130], VMir [131], and miRNAFold [116]. These methods do not take into account sequence homologies and similarities to known miRNAs, but they predict putative pre-miRNAs using the features of pre-miRNA sequences and their secondary structures [132]. Ab initio methods produce more false positives than the other prediction methods described [133] that have a greater discriminative power since they use homology and evolutionary conservation as filters. Therefore, the primary challenge in ab initio methods is the selection of appropriate parameters/features to achieve the elimination of false positive predictions, for instance, sequence features such as minimum free energy (MFE), base pairing propensity, GC and GU content, number and size of bulges, and hairpin loop length [132]. Actually, there is no single parameters/features combination; each algorithm uses its own strategy to distinguish between true and false pre-miRNAs.

RNAFold (Vienna RNA package) [128] and mfold (UNAFold package) [99] are usually used as a preliminary step in ab initio algorithms to predict RNA secondary structures from the genome sequence of interest (both tools are also available online and downloadable) [131]. Furthermore, the mfold web server has been used directly to predict the pre-miRNA-like hairpin structures encoded by the HBV genome [69,70].

The use of machine learning (ML) algorithms allows the features from known pre-miRNAs to be extracted and uses them as the training set. The widely used algorithms are based on supervised classification, such as support vector machine (SVM), random forest (RF), k-nearest neighbor (KNN), and Naïve Bayes (NB) [80].

The choice of positive and negative examples is crucial in the building of a classifier. Known pre-miRNAs (e.g., from miRBase) and random sequences or pseudo hairpins or other RNA structures (tRNA, rRNA, and mRNA) are commonly used as positive and negative datasets, respectively [134]. To date, the number of known/real pre-miRNAs is much smaller than the negative samples generated. This imbalance is even more pronounced when considering viral miRNAs due to the limited annotations of virus pre-miRNAs. Moreover, it is difficult to create a good negative dataset [133,134,135]. 

Despite the critical issues that characterize these computational algorithms, some interventions and novel strategies are starting to improve the specificity and accuracy of these predictive models (to avoid false positive predictions), especially for viruses. One approach might be to use only known viral pre-miRNAs as positive examples and by extracting features with specific tools (e.g., miRNAfe [136]) [137], or by adding features that are based on the mature miRNA [138]. 

Vir-Mir db [129,130] is a specialized database in candidate miRNA hairpins from viral genomic sequences. The web interface is user-friendly, and the data can be queried through the hierarchical menu or the search function (GenBank identifier or RefSeq accession number is recommended). Some information available are strand, start position, length, loop score, MFE, as well as target prediction from RNAhybrid [139].

VMir v.1.5 [131] remains the most used software for the identification of novel viral miRNAs by pre-miRNA sequence prediction. This bioinformatic tool uses RNAFold to predict the secondary structures and it selects the putative pre-miRNAs by calculating a score for the hairpins produced. The VMir package contains two freely-available tools: the VMir Analyzer and VMir Viewer. The first allows us to analyze and predict putative miRNAs from the genome input file, such as GenBank, FASTA, and raw text files. The results can be visualized, filtered, and exported by VMir Viewer [140]. 

Another ab initio software used to predict the secondary structure is miRNAFold [116]. The algorithm used allows us to speed up the search of pre-miRNAs in every genomic sequence. miRNAFold is user-friendly and available online.

The potential pre-miRNAs identified by precursor prediction tools are then analyzed to extract the corresponding mature miRNAs. MatureBayes [141] and MaturePred [142] are some of the bioinformatic tools used to find mature miRNA within the pre-miRNAs structures. MatureBayes is a probabilistic algorithm that uses a Naïve Bayes classifier. It is a web-based tool, and the search can be performed by pasting the sequence of a pre-miRNA. MaturePred is a web tool as well and it is designed for prediction mature miRNAs using SVM. The positive dataset used is constructed by real miRNA duplexes from miRBase.

**Table 2:** Overview of described microRNA identification tools.

Database/Tool Name	Summary Features	Link	Refs.
MiRscan	Comparative genomic approach for pre-miRNA sequence.	http://hollywood.mit.edu/mirscan/index.html (accessed on 1 June 2023)	[124,125]
RNAFold	Predict secondary structure of single-stranded RNA or DNA sequences using minim free energy (MFE).	http://rna.tbi.univie.ac.at/cgi-bin/RNAWebSuite/RNAfold.cgi (accessed on 2 August 2023)	[128]
mFold	Ab initio method for predicting RNA secondary structure sequence using minimum free energy (MFE).	http://www.unafold.org (accessed on 9 May 2023)	[99]
Vir-Mir db *	Database for candidate hairpins from viral genomic sequences. Target prediction tool from RNAhybrid.	http://alk.ibms.sinica.edu.tw (accessed on 30 April 2023)	[129,130]
VMir *	Software for identification of novel miRNAs by pre-miRNA sequence prediction.	http://virus-genomics.org/software/vmir/vmir.html (accessed on 27 May 2023)	[140,143]
miRNAFold	Ab initio method for predicting pre-miRNA sequence.	https://evryrna.ibisc.univ-evry.fr/miRNAFold (accessed on 15 July 2023)	[116,144]
MatureBayes	Prediction of mature miRNA using Naïve Bayes classifier	http://mirna.imbb.forth.gr/MatureBayes.html (accessed on 9 June 2023)	[141]

* Viral specific database/tool.

### 3.3. Target Prediction

The differential expression of miRNAs (DE-miR) refers to comparing miRNA levels between different biological conditions, such as healthy and pathological states or across different disease stages. This comparison aids in the identification of miRNAs that are upregulated (increased expression) or downregulated (decreased expression) in the condition of interest.

After the identification of these miRNAs, the second step is to investigate their role in gene expression regulation by their relative gene targets. In fact, the identification of the miRNA targets is essential for understanding their functional roles and unraveling the regulatory networks and biological pathways influenced by miRNAs and their targets (i.e., mRNA).

Bioinformatics approaches, such as sequence complementary analysis and computational algorithms, are employed to predict potential miRNA target sites in mRNA sequences. These predictions are an alternative means of investigating and can support the experimental procedures.

The miRNA *seed sequence* is one of the main miRNA features used in target prediction algorithms, along with thermodynamic stability/free energy, evolutionary conservation, target site abundance, and accessibility of the target site [145]. Moreover, as with identification tools, ML algorithms have been developed for predicting novel targets. In fact, the ML strategy allows for the extraction of features from known and validated miRNA-mRNA interactions, which are used as a training dataset (positive dataset). However, the critical point is again the quality of positive and negative datasets that affect the training process, and similarity between datasets can lead to underfitting or overfitting [81].

The main problem with target prediction tools remains the high rate of false positives, and because of that, the combination of several tools with different strategies can improve predictions [146,147]. Due to some limitations of the current available algorithms, the use of different tools is necessary to predict and discover novel host–virus target interactions. Currently, algorithms are adapted almost exclusively for human miRNAs.

In recent years, many bioinformatic tools and algorithms about target predictions have been developed, but the broadly used ones remain the state-of-the-art tools, including TargetScan [148,149], miRDB [150,151], RNAhybrid [152], and RNA22 [153]. Furthermore, these tools have a strong impact on the development of recent target prediction tools and algorithms [91], such as miRTarBase [93], DIANA-TarBase [94], miRDB [151], MirDIP [111], and mirTar/mirTarP [92] (Table 3). 

TargetScan [148,149] is one of the first target prediction tools and is updated regularly by adding novel features in prediction methods (e.g., different position of the target site). TargetScan predicts target genes in various species, including humans and other animals. This prediction is achieved through the degree of sequence complementary between the miRNA and target, as well as the presence of conserved miRNA binding sites within 3′ UTR of the target mRNA. Moreover, TargetScan provides a ranking of the predicted target gene, i.e., context ++ and Aggregate P_CT_ scores. The context ++ score is calculated for each specific site as the sum of the contribution of 14 features [154]. Meanwhile, P_CT_ scores are the “Probability if Conserved Targeting” calculated for highly conserved miRNA families [155]. TargetScan may lose some targets by not considering mismatches and non-canonical target regions [29,147]. The target search is conducted by miRNA name.

miRTarBase [93,156] is a database of experimentally validated miRNA–target interactions (MTIs). In the last version, 2,200,449 verified MTIs and 27,172 target genes from 37 species have accumulated. This tool is regularly updated to include new MTIs and provides annotations and information about miRNA and target gene identifiers, the experimental method used, and the related publication. The search can be performed by miRNA name, target genes, disease/pathways, and species. To date, some viruses (e.g., Epstein–Barr and herpes B virus) are available, but not for hepatitis. In the context of HBV, for example, it is possible to search using hepatitis B disease.

DIANA-TarBase [94] is another target database containing experimentally validated MTIs. Similar to miRTarBase, targets may be searched by name of the miRNA or gene. In addition, species, method, validation, cell, and tissue type can be added as search filters. In the last version of TarBase v.8, 1,080,278 entries and 665848 miRNA-mRNA interactions are available. DIANA-TarBase is also included in DIANA-microT, a webserver tool used to computationally predict miRNA-mRNA interactions. In the 2023 version, predicted targets of virally encoded miRNAs have been included [157]. Moreover, this tool predicts the miRNA binding site in both 3′ UTR and CDS of the transcripts and it incorporates data from numerous resources, such as miRBase [74], MirGeneDB [158,159], and HMDD [96]. In this case, too, the search is performed by miRNA and/or gene.

A web-based tool to search predicted targets by miRNA and mRNA sequence is miRDB [150,151]. miRDB allows us to custom each miRNA target search by sequence and non-canonical target site (i.e., coding region or 5′ UTR), as well as via miRNA and mRNA name. In addition, miRDB also presents target expression profiles in specific cell lines and information about pre-miRNA sequence and functional annotations. In the context of novel/unknown miRNA or target, searching through sequence may aid in discovering and understanding the novel putative interaction.

MirDIP (microRNA Data Integration Portal) [160] was born as an miRNA–target predictions database that aggregates miRNA information from multiple databases and target prediction tools, such as miRBase [88], MirGeneDB [158,159], GEO [84], DIANA [161], miRDB [150], RNA22 [153], and Targetscan [148,149].

The latest version 5.2 [111] includes miRNA and gene expression data in different tissue and disease contexts. Moreover, it provides regular new releases by integrating novel miRNA sequences directly from publications. In the context of viral diseases, it might be used for selecting deregulated miRNAs and their targets inside specific tissues and disease conditions, such as blood, hepatocytes, immune system, and liver cirrhosis. Furthermore, predicted targets of miRNA may be obtained from the online tool via unidirectional (gene or miRNAs) and bidirectional (gene and miRNAs) searches. MirDIP is a web-based tool, but API are available for Phyton, R, and Java.

As with other types of bioinformatic tools, specific tools for viruses and relative diseases are insufficient. mirTar/mirTarP [92] is one of the few tools that consider human–virus interspecies miRNA target genes to be computationally predicted. In particular, mirTar considers viral miRNA targets on the human genome and human miRNA targets on the virus genome. Viruses are classified according to the International Committee on the Taxonomy of Viruses (ICTVs) category. For example, mirTar can be queried by virus name or the ICTVs category to facilitate the search. The mirTarP tool has been developed to bridge the gap between different species (i.e., human/host and viruses), combining Blast, RNAhybrid, and RNAfold algorithms to predict targets. The mirTarP algorithm contains two parameters: base match and the minimum free energy between miRNA and the target and can be queried using miRNA and the target gene sequence as the input files. The advantage of this target prediction tool is that it works independently from conservation to find targets on virus genomes. To date, in mirTar, there are 3148 predicted gene targets for HBV.

**Table 3:** Overview of described microRNA target prediction tools.

Database/Tool Name	Summary Features	Link	Ref.
TargetScan	Host (human) miRNAs target gene. Computationally predicted.	https://www.targetscan.org/vert_80/ (accessed on 3 August 2023)	[148]
miRTarBase	Host (human) miRNAs target gene. Disease/pathways. Target expression. Host (human) miRNA expression. Experimentally validated	https://mirtarbase.cuhk.edu.cn (accessed on 11 April 2023)	[93]
DIANA-TarBase	Host (human) miRNAs target gene. Disease/pathways. Target expression. Experimentally validated and computationally predicted.	http://www.microrna.gr/tarbase (accessed on 12 June 2023)	[94]
miRDB	Host (human) miRNAs target gene. Computationally predicted.	http://www.mirdb.org (accessed on 18 August 2023)	[150]
MirDIP	miRNA target prediction database. Experimentally validated and computationally predicted.	https://ophid.utoronto.ca/mirDIP/ (accessed on 4 April 2023)	[111]
mirTar/mirTarP *	Human–virus interspecies miRNAs target gene. Computationally predicted.	https://mcube.nju.edu.cn/jwang/mirTar/docs/mirTar/ (accessed on 20 May 2023)	[92]

* Viral specific database/tool.

### 3.4. Functional Enrichment Analysis and microRNA–Disease Associations Tools

Given the crucial role of miRNAs in post-transcriptional gene regulation, computational analysis can aid in investigating and identifying the biological functions, pathways, and processes that are significantly associated with a set of miRNAs. Therefore, the functional enrichment analysis provides information about the role of miRNAs in different biological processes and diseases. In the context of HBV infection and its related diseases, it is important to understand and discover the pathways in which the virus is involved. Some miRNAs are associated with pathways related to HCC, apoptosis, immune response, and signaling, for instance. 

Functional analysis was firstly devoted to discover gene-related biological processes, molecular functions, and cellular components by exploiting Gene Ontology (GO) [162,163] and pathways by the Kyoto Encyclopedia of Genes and Genomes (KEGG) [164], which still remain well-established resources for functional enrichment tools. GO [163] is a curated database and vocabulary that allows us to annotate and describe function genes and their products. GO terms are organized in three main categories: biological function, molecular function, and cellular component. Therefore, GO enrichment analysis is used to determine which GO terms are overrepresented, starting from the gene set of interest. KEGG [164] is a specialized and curated pathways database. These pathways describe complex biological processes as interaction networks, where genes and proteins within pathways are linked to each other in cascade events that can determine a particular function and/or involvement in diseases. There are several pathways in KEGG, covering a wide range of biological processes, including metabolism, cellular signaling, immune system, and disease. Several species are included, such as humans, viruses, plants, and animals. GO and KEGG resources are often integrated into tools for miRNA pathway/enrichment identification and interaction network analysis [165,166,167,168]. The discussed tools are listed in Table 4.

There are different strategies to analyze miRNA functions by predicting miRNA target genes or using miRNA annotations databases [169]. The most used is by predicting the target genes of the selected miRNAs and performing enrichment analysis through statistical tests (e.g., hypergeometric or Fisher’s exact tests). After the identification of gene targets, the generated list of genes can be used to perform functional enrichment analysis through several bioinformatic tools, such as DAVID [170], Enrichr [171], and FunRich [168], which queries a different library and annotations database, including GO and KEGG.

DAVID is among the most popular web server tools for functional annotation and enrichment analysis. It allows us to analyze and extract biological meaning from large gene lists by mapping genes to the associated biological annotations, such as gene ontology terms [172]. In the last update, several novel annotation types have been added, including drug–gene interactions, gene–disease annotations, and pathways [173].

Enrichr [171] is a web server and an R package enrichment tool which contains a vast number of gene set libraries, including KEGG, GO, transcription factors, and disease databases (e.g., DisGeNet [174]). 

FunRich v.3.1.4 [168] is a free software developed for gene and miRNA enrichment. Moreover, the tool provides and customizes graphical representations, including Venn diagrams, heatmaps, pie charts, and bar graphs. In addition, target prediction and ID conversion for genes and miRNAs can be performed.

To date, several tools specifically dedicated to miRNA-pathway analysis have been developed (e.g., DIANA-miRPath v4.0 [175] and MIENTURNET [165]), where the prediction of gene targets and relative enrichment analysis is performed. 

DIANA-miRPath v4.0 [175] is an online functional analysis of miRNAs and related pathways tool. It is designed to predict the potential biological pathway that miRNAs may be involved in based on their target genes. Inside the tool, the target genes are predicted from other resources, such as DIANA-TarBase, TargetScan, and miRTarBase. DIANA-miRPath v4.0 offers multiple statistical methods (e.g., Fisher’s exact test, EASE score, and False Discovery Rate) to perform enrichment analysis.

MIENTURNET (MicroRNA ENrichment TURned NETwork) [165] has been developed to explore and analyze miRNA–target interactions by combining statistical analysis (i.e., hypergeometric test and False Discovery Rate) and network theory. Computationally predicted gene targets are acquired from TargetScan, while those are experimentally validated from miRTarBase. This web tool also allows us to perform functional enrichment analysis of miRNA–target genes by querying annotation pathway databases such as KEGG, Reactome [176], WikiPathways [177], and Disease Ontology [178].

Specialized databases have been developed to collect miRNA–disease associations from the scientific literature and research articles, including DisGeNET, the Human microRNA Disease Database (HMDD), RNADisease v4.0, and miRwayDB. 

DisGeNET [174,179] is a comprehensive and updated resource that contains gene–disease associations and variants but also includes miRNAs. The information is integrated from the scientific literature. The search can be performed by diseases, genes, and variants. This tool combines the functions of Cytoscape and data from DisGeNET [180].

The Human microRNA Disease Database (HMDD) [96] is a database used to explore experimentally validated associations between miRNAs and human diseases by providing information about miRNAs implicated in several diseases. All data come from the scientific literature and research articles. Therefore, each entry is curated and annotated. The database is web-based and allows us to browse, search, and download data.

RNADisease v4.0 [181] is a repository for RNA–disease associations, including host (human) and viral miRNAs. This resource integrates several data from RNA sequences (e.g., TCGA), the literature, experimentally validated databases, and prediction algorithms. Searching can be performed using RNA ID/Symbol or Disease name (Disease Ontology, MeSH vocabularies, and KEGG disease terms). Computational prediction and strong or weak experimental evidence can be used to filter searches. In addition, it is possible to explore miRNA–disease association by browsing directly within the following categories: disease, RNA category, species, and predicted algorithm. RNADisease is user-friendly and offers several tools for disease enrichment, RNA–disease prediction, and cancer analysis, by adding a list of miRNAs.

A complete database to fill the gap between miRNA dysregulation and disease-associated pathways is miRwayDB [166]. In fact, miRwayDB has been developed to investigate miRNA-pathway associations in pathophysiological conditions. This database collects only experimentally validated information from the literature. Data about disease, associated miRNAs, experimental sample type, the regulation pattern of miRNA, pathway associations, and targeted members of dysregulated pathways are available in the search results. Additionally, miRwayDB assigns scores to pathways, genes, and miRNAs to evaluate the resulting miRNA–gene–pathway–disease axis.

**Table 4:** Overview of described miRNA functional enrichment, pathway, and disease association tools.

Database/Tool Name	Summary Features	Link	Ref.
DAVID	Gene functional annotation. Gene enrichment analysis.	https://david.ncifcrf.gov (accessed on 11 April 2023)	[173]
Enrichr	Webserver with data from several databases. Gene enrichment analysis.	https://maayanlab.cloud/Enrichr/ (accessed on 18 July 2023)	[171]
FunRich	Gene enrichment. miRNA enrichment. Target prediction. ID conversion.	http://funrich.org (accessed on 22 June 2023)	[168]
DIANA-miRPath v4.0	miRNA functional analysis. Predict pathways.	http://www.microrna.gr/miRPathv4 (accessed on 5 July 2023)	[175]
MIENTURNET	miRNA–target interactions. miRNA functional enrichment.	http://userver.bio.uniroma1.it/apps/mienturnet/ (accessed on 22 May 2023)	[165]
DisGeNET	Gene–disease associations. Gene variant–disease associations.	https://www.disgenet.org (accessed on 19 April 2023)	[180]
HMDD	miRNA–disease associations. Experimentally validated.	http://www.cuilab.cn/hmdd (accessed on 29 August 2023)	[96]
RNADisease v4.0	RNA–disease associations. Experimentally validated.	http://www.rnadisease.org (accessed on 2 May 2023)	[181]
miRwayDB	miRNA–disease pathway associations. Experimentally validated.	http://www.mirway.iitkgp.ac.in (accessed on 19 June 2023)	[166]

## 4. microRNAs and Hepatitis B Virus

The liver plays a vital role as a primary blood-filtering organ, making it susceptible to blood-born infections. Among these infections, several viruses can infect the liver. The most common viruses are part of the subgroup of hepatitis viruses, which includes hepatitis A virus (HAV), hepatitis B virus (HBV), hepatitis C virus (HCV), hepatitis D virus (HDV), and hepatitis E virus (HEV). HBV, HCV, and HDV could induce hepatocellular carcinoma (HCC). Therefore, the presence of these viruses represents a major risk factor for the development of HCC, especially HBV [182].

Persistence infection of HBV can lead to chronic hepatitis B (CHB), triggering an immune response and causing genetic damage and constant oxidative stress. If left untreated, CHB can progress to fibrosis, cirrhosis, and finally to HCC.

HBV is able to integrate into the host genome by inducing mutations in cancer-related genes (e.g., tumor suppressor genes or proto-oncogenes) and causing epigenetic changes in the liver [183]. In fact, it has been observed that the risk of developing HCC increases proportionally with the HBV viremia [184].

The identification and characterization of viral/host miRNAs and their gene targets can aid in understanding the complex interactions between viruses and their host. Consequently, they might serve as diagnostic biomarkers based on their presence and levels in patient samples [185,186,187], offer insights into disease prognosis through their different expression during infection [188], and present opportunities as therapeutic targets for the development of antiviral drugs (i.e., anti-miR therapy) [189,190]. Furthermore, their use could extend to monitoring treatment response by evaluating the treatment efficacy [191].

**Figure 1:**
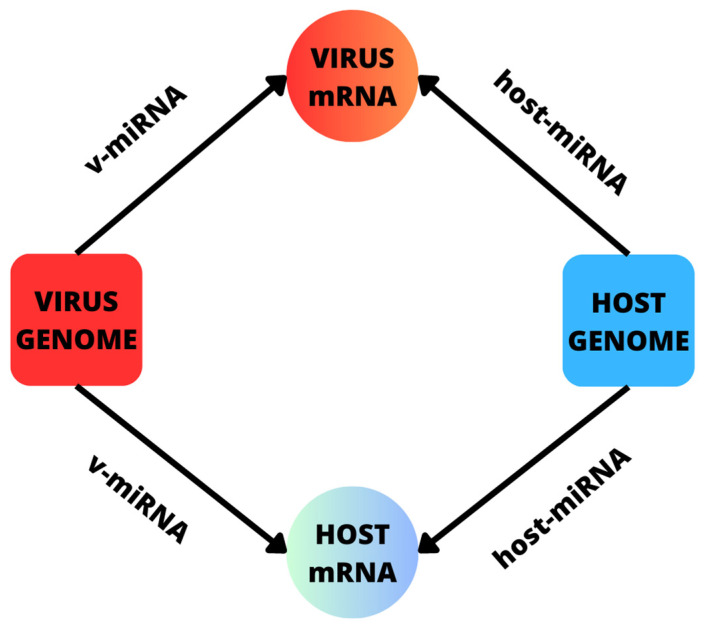
The interplays between viral and host (human) miRNAs during viral infection.

The dynamic of miRNAs during HBV infection is complex because the presence of the virus can generate an aberrant expression of miRNAs. Moreover, a specific miRNA can bind several mRNAs, while a single mRNA can serve as a target for various miRNAs. As a consequence, host-miRNAs can bind viral mRNAs, as well as their own mRNAs [192,193]. The same applies to v-miRNAs, which can regulate host and viral mRNAs (see Figure 1) [113,194,195].

In general, host-miRNAs could be divided in pro-viral and antiviral based on their actions [77] (a brief summary of the mentioned miRNAs is shown in Table 5). Pro-viral host-miRNAs create a more favorable environment for viral replication and dissemination by suppressing antiviral factors. Meanwhile, antiviral host-miRNAs are part of the host defense by targeting the viral components or host factors involved in viral replication or the immune response pathway [196].

Among the HBV viral proteins, there is evidence that the HBx protein plays a crucial role in miRNA expression through epigenetic modifications [197]. HBx protein can interact with transcription factors (e.g., JAK/STAT pathway) and transduction pathways (signaling pathways) [198]. Furthermore, several studies have shown that HBx protein is able to dysregulate host-miRNAs by inhibiting the miRNA biogenesis process, such as the Drosha/Dicer mechanism [199,200]. Therefore, HBx can act as an miRNA sponge. HBx can increase the miRNA expressions by the DNA hypomethylation of the miRNA promoter. Consequently, HBx also plays a crucial role in the pathogenesis of HBV-related HCC [197,201].

By targeting host genes, v-miRNAs can regulate and affect apoptosis, innate and adaptive immunity, cell growth, and differentiation [202,203]. Furthermore, they can also modulate various aspects of viral replication, including immune evasion, latency establishment, and viral pathogenesis [204].

It is along these lines that a part of this research is focusing on identifying which miRNAs are dysregulated in different biological pathways and disease stages. Among the biological pathways, the regulation of HBV replication, modulation of host immune response, and apoptosis have been the most studied. Meanwhile, CHB, fibrosis/cirrhosis, and HBV-related HCC have been the most compared diseases.

About HBV replication, several studies have shown that some miRNAs are associated with viral replication, such as miR-802, which targets the SWI/SNF-Related Matrix-Associated Actin-Dependent Regulator of Chromatin Subfamily E Member 1 (SMARCE1). HBV DNA replication, HBsAg, and HBeAg were increased following the overexpression of miR-802 in HepG2.2.15 cells [205]. It has also been shown that the HBV replication in HepG2.2.15 cells was promoted by the downregulation of miR-101, which has among its targets Forkhead box protein O1 (FOXO1). FOXO1 was considered an activator of HBV transcription and a tumor suppressor in HCC. Therefore, the upregulation of FOXO1 can increase viral replication [206]. Other research suggests that miR-501 is a potential therapeutic target because it may induce HBV replication by targeting HBXIP (hepatitis B X-interacting protein), an inhibitor of HBV replication [207]. Meanwhile, miR-199a-3p and miR-210 may suppress viral replication by targeting HBsAg and HBV pre-S1, respectively [208].

A recent study has explored the role of miRNAs in CHB progression by the regulation of covalent closed circular DNA (cccDNA) in liver tissues from CHB-GZ patients. The results have shown that miR-4295 and Zinc Finger Protein 224 (ZNF224) may be a regulatory axis for intrahepatic cccDNA by the downregulation of this miRNA. In fact, the most enriched biological processes were about the regulation of the viral life cycle, viral process, and viral genome replication by analysis of the KEGG and GO terms [209]. Similar to other studies, it is essential to validate these findings both in vivo and in vitro experiments, while also considering the inclusion of larger and more diverse clinical cohorts.

Another study has suggested that miR-302c-3p may have anti-HBV activity by reducing HBsAg production by lowering the cccDNA levels and inhibiting core protein production. The study was performed using HepG2-hNTCP-C4 cells; therefore, additional studies are required [210].

miR-125a is one of the most interesting miRNAs because it is able to bind HBsAg, and because it correlates with HBx. miR-125a was found to be overexpressed in the liver with a high HBV DNA level in miR-125a-inhibited HBsAg translation, and it may be considered an antiviral host-miRNA. Moreover, the research suggested that the HBx-miR-125a-HBsAg interaction network allows for the HBV to escape the immune system and lead to CHB [211].

In a recent study, HBx upregulated miR-203; as a result, miR-203 downregulated BANF1 and the viral titer was increased in Huh7, Hep G2, and Hep G2.2.15 cells [212]. In other studies, the HBV DNA levels were increased after the overexpression of miR-144-5p and miR-375 in HepG2.2.15 and HepAD38 cells [46].

A good biomarker must meet certain criteria, including high accuracy, reproducibility, early detection, quantifiability, cost-effectiveness, as well as non-invasiveness (e.g., blood/plasma, urine, or saliva). Therefore, different miRNA levels have been researched between different disease stages [188]. One of these studies showed that miR-122, miR-22, miR-99a, and miR-125b were upregulated in the serum of CHB patients compared to healthy patients, suggesting that serum miRNA profiles could aid in monitoring liver disease progression. It is interesting to note that miR-125b has been associated with viral replication. Therefore, high levels of this miRNA may indicate a more active or aggressive phase of HBV infection [185].

miR-122 has also been studied in one main study investigating serum levels from CHB patients. The serum miR-122 levels were found to correlate with HBV DNA and HBsAg levels (patients with higher HBV DNA and HBsAg levels had significantly higher miR-122 levels), indicating a relationship with viral replication and suggesting miR-122 as a biomarker for risk stratification in HBV patients. Moreover, miR-122 may also have therapeutic potential as it is involved in hepatotropic viruses and hepatocyte differentiation [213].

Among various miRNAs associated with liver cirrhosis in CHB patients, miR-29 has demonstrated a strong negative correlation with the progression of cirrhosis and fibrosis, as well as necroinflammation grades. This is significant because cirrhosis primarily develops due to the accumulation of collagen deposited by hepatic stellate cells (HSCs) within the extracellular matrix of liver cells. Elevated levels of miR-29 may suppress cirrhosis and fibrosis through gene targets that are involved in TGF-β and NF-κB signaling pathways. In addition, the overexpression of miR-181b, miR-214-5p, and miR-221/222 has been associated with the progression of liver fibrosis by the activation of HSCs [193].

A comprehensive review investigated and summarized the dysregulation of miRNAs by HBx in HBV-HCC and immune pathways. For example, miR-155, miR-17-19 family, miR-21, miR-29a/b, and miR-221/222 were upregulated by HBx, and the most important relative gene target is the tumor suppressor gene Phosphatase and Tensin Homolog (PTEN). In fact, PTEN helps to control cell division and apoptosis; thereby, losing PTEN function can lead to uncontrolled cell growth and contribute to the development of cancer [200].

HBx can downregulate miR-132 involved in the Akt signaling pathway, leading to the proliferation of hepatoma cells in HBV-related HCC [214]. Likewise, Let-7 and miR-122 have been found to be downregulated by HBx; these miRNAs are associated with cell proliferation and tumor growth by targeting signal transducer and the activator of transcription 3 (STAT3) and cyclin G1, respectively [197].

It has been investigated that miR-137 can be downregulated by HBx and consequently increase HCC cell proliferation by targeting Neurogenic locus notch homolog protein (NOTCH1) [215].

Different expressions of miRNAs can also be used to evaluate the effect of an antiviral treatment. In fact, it has been shown that miRNA levels vary in response to antiviral treatment. For example, miR-3960, miR-126-3p, and miR-335-5p have been investigated as potential biomarkers to predict HBsAg clearance by comparing the response of PEG-IFNα-2a treatment in CHB patients. The expression of these miRNAs was downregulated in the response group compared to the non-response group, and their target gene pathways included signaling pathways [216].

miR-122-5p, miR-99a-5p, and miR-192-5p are considered liver-specific miRNAs (e.g., miR-122 represents more than seventy percent of all the liver miRNAs), and thus they are the most studied miRNAs in liver diseases. These three miRNAs have been used for the prediction of sustained virologic response (SVR). They are overexpressed in CHB and inactive carriers (ICs) and are downregulated during and after PEG-IFNα treatment in SVR [217]. Moreover, the combination of miR-122-5p, miR-99a-5p, miR-192-5p, miR-126-3p, miR-335-5p, and miR-320a has been used as a biomarker (called MiR-B-Index) to identify IC patients. This MiR-B-Index has differentiated between active and inactive HBV infections and may aid in the identification of patients responsive to PEG-IFNα.

Still, another study demonstrated that the serum miR-192-5p level was correlated with the levels of HBV DNA, HBsAg, and hepatitis B core-related antigen in CHB patients treated with PEG-IFNα-2a. The levels of miR-192-5p have remained high in non-virological response patients compared to responders after 24 weeks of treatment [218]. These results suggest that miR-192-5p may be used as a predictor of the therapeutic efficacy of PEG-IFNα.

To date, only two HBV-encoded miRNAs have been identified, i.e., HBV-miR-3 and HBV-miR-6 (in the process of validation). HBV-miR-3 is encoded from nucleotides 373 to 393 in the HBV genome and was generated from 3.5-kb, 2.4-kb, and 2.1-kb HBV. It has been identified in HBV-positive HCC tissue and it is generated from PreC, PreS1, and PreS2 mRNAs. HBV-miR-3 was able to reduce HBV replication and protein expression by inhibiting HBc protein expression [69]. In addition, it has been shown that HBV-miR-3 can activate the JAK/STAT signaling pathway, a key component of the immune response, by downregulating the Suppressor of Cytokine Signaling 5 (SOCS5). The activation of the JAK/STAT pathway allows us to enhance the IFN-induced anti-HBV response. Therefore, this mechanism could reduce acute liver cell injury and affect persistent HBV infection [113].

HBV-miR-3 was found to be overexpressed during HBV infection; consequently, it may inhibit the translation of its target protein phosphatase 1A (PPM1A) and promote cell proliferation [194]. Moreover, another important target of HBV-miR-3 is the tumor suppressor PTEN, which is downregulated in HCC. The downregulation of PTEN expression can inhibit apoptosis and increase HCC invasion [219]. In another study, HBV-miR-3 was not detected in the plasma and liver tissue of CHB patients using NGS [70].

In a recent study, HBV-miR-6, a putative novel HBV v-miRNA, has been identified in liver tissue from CHB patients. The HBV-miR-6 expression level was correlated with hepatic HBV DNA and plasma HBsAg levels. However, the overexpression of HBV-miR-6 had no relevant effect on HBV replication. Further research is needed to elucidate the specific mechanisms and functions of HBV-miR-6 in HBV infection [70].

**Table 5:** Overview of miRNAs involved in HBV infection.

microRNA/Deregulation	Function	Gene Target	Refs.
hsa-miR-802 ↑	Promote viral replication.	SMARCE1	[205]
hsa-miR-101 ↓	Promote viral replication.	FOXO1	[206]
hsa-miR-501 ↑	Promote viral replication.	HBXIP	[207]
hsa-miR-203 ↑	Promote viral replication.	BANF1	[212]
hsa-miR-125b ↑	Promote viral replication.	SCNN1A, LIN28B	[185,193,220]
hsa-miR-210 ↑	Suppress viral replication.	HBV pre-S1	[208]
hsa-miR-4295 ↓	Suppress viral replication.	ZNF224	[209]
hsa-miR-302c-3p	Suppress viral replication.	BMPR2, HNF4A	[210]
hsa-miR-125a ↑	Suppress viral replication.	HBsAg	[211]
hsa-miR-122 ↑	Suppress viral replication.	HBV-RNA, CCNG1	[185]
hsa-miR-199a-3p ↑	Suppress viral replication.	HBsAg	[208]
hsa-miR-29 ↑	Promote viral replication. Progression of fibrosis/cirrhosis.	TNFAIP3, SMARCE1, PTEN	[193,221]
hsa-miR-181b ↑	Progression of fibrosis/cirrhosis.	-	[193]
hsa-miR-214-5p ↑	Progression of fibrosis/cirrhosis.	-	[193]
hsa-miR-221/222 ↑	Progression of fibrosis/cirrhosis.	-	[193]
hsa-miR-137 ↓	Promote cell proliferation in HBV-related HCC.	NOTCH1	[215]
hsa-miR-22 ↓	Tumor suppressor HBV-related HCC.	CDKN1A, CDK6, SIRT1	[185]
hsa-miR-192-5p ↑	Correlation with virological response.	-	[217,218]
HBV-miR-3 ↑	Suppress viral replication. Promote cell proliferation in HBV-related HCC.	HBV-RNA, SOCS5, PPM1A, PTEN	[69,113,194,219]

↑ Upregulated miRNA; ↓ downregulated miRNA.

## 5. Conclusions

The dynamic and role of miRNAs during HBV infection remain unclear due to the dependence on the genotype of the virus and variability among host and miRNA involved, but also especially due to the lack of comprehensive specific bioinformatic tools and a solid model for the experimental validation.

The scarcity of specific viral miRNA and virus–host interaction databases and tools can present challenges to researchers. Comprehensive and up-to-date repositories are crucial for advancing the understanding of virus–host interactions and the role of viral/host miRNAs on cellular processes. To date, many databases and tools are not continuously updated, and this type of information is often unclear and hard to access. Infrequent updates may lead to the loss of recently identified miRNAs and their target genes, lack of information on changes in existing miRNA functions, as well as not covering all relevant aspects, such as connections between miRNAs and diseases or pathways. To avoid these limitations, it is essential to establish efficient, frequent, and clear updates.

As the field of virology and miRNA research progresses, it is expected that more efforts will be made to establish comprehensive databases and specific bioinformatic tools, thereby bridging the gap between human and viral miRNA resources.

Developing specialized databases and bioinformatic tools for viral/host miRNAs related to infection diseases may aid in the development of antiviral strategies and therapeutics and the discovery of potential biomarkers. However, bioinformatics can generate a large number of miRNA data, and not all of them may be biologically relevant. In addition, several algorithms have been developed by using experimental data, which may introduce some bias due to the lack of a rigorous, schematic, and standardized experimental protocol. Therefore, improving the accuracy and specificity of algorithms is necessary [80,134,138].

The use of various cohorts of patients in different disease stages (CHB, HBV/HDV coinfection, fibrosis/cirrhosis, and HBV-related HCC), different samples (e.g., cells, plasma/serum, and blood), and the choice of baseline characteristics (e.g., level of liver damage and viral replication markers as comparison variables) are critical aspects of the experimental method. Moreover, most of the studies involve Asian populations [222], and this may introduce bias due to the lack of population heterogeneity. In addition, clinical trials on CHB and HBV-related fibrosis/cirrhosis are still limited compared to HBV-related HCC. This is especially true for the search for new potential miRNA biomarkers. Indeed, the exploration for novel miRNA biomarkers involves several different stages that integrate biology, bioinformatics, experimental validation, and clinical research. Therefore, the challenges faced also include the standardization of measurement platforms (e.g., Northern blot, real-time RT-PCR, microRNA microarray, and next-generation sequencing), normalization control strategies, such as the addition of a synthetic miRNA or a reference gene, and the sample processing method [223] in order to make studies reproducible and comparable.

To date, the most frequently reported dysregulated miRNAs in HBV-related HCC include miR-21, miR-29a/b, miR-122, miR-125a/b, miR-155, miR-199, and miR-221/222 [222,224]. miR-22, miR-99a, miR-122, and miR-125b have been suggested as prognostic biomarkers in liver disease progression during HBV infection [185]. In addition, miR-29, miR-181b, and miR-221/222 have demonstrated a correlation with the progression of fibrosis and cirrhosis [193]. Meanwhile, miR-126-3p and miR-192-5p may be used to evaluate the antiviral treatment response [216,218].

As shown by the studies cited in this review, other critical issues concern target and functional redundancy because miRNAs often have multiple target genes. Moreover, miRNA expression is usually tissue-specific, but the same miRNA could have different functions, and therefore show aberrant expression in different types of disease [38]. Consequently, identifying the precise role of a specific miRNA is complex, representing one of the main challenges [37]. The abovementioned host (human) miRNAs are indeed also implicated in other diseases and functions not related to HBV (e.g., miR-122 in HCV) [40,187]. In this context, it makes even more sense to focus more on the study of viral miRNAs, which may show greater sensitivity and specificity.

Despite these limitations, miRNA analysis remains an interesting area of research with great potential in the clinical management of viral infections, from diagnosis and prognosis to personalized treatment and the development of innovative therapeutic approaches.

## Data Availability

Not applicable.
